# Understanding the Impact of Childhood Sexual Abuse on Men’s Risk Behavior: Protocol for a Mixed-Methods Study

**DOI:** 10.2196/resprot.9071

**Published:** 2018-02-26

**Authors:** Martin J Downing Jr, Dominique Brown, Jeffrey Steen, Ellen Benoit

**Affiliations:** ^1^ Public Health Solutions New York, NY United States; ^2^ National Development and Research Institutes, Inc New York, NY United States; ^3^ School of Social Work Bridgewater State University Bridgewater, MA United States

**Keywords:** protocol, childhood sexual abuse, MSM

## Abstract

**Background:**

Childhood sexual abuse (CSA) remains a critical public health issue among black and Latino men who have sex with men (MSM), as it is associated with multiple negative outcomes including substance misuse, poor mental health, revictimization, and high-risk sexual behavior. Most CSA research with MSM relies on quantitative assessment that often precludes consideration of cultural variations in how formative sexual experiences are understood and is based on inconsistent or overly restrictive definitions of abuse, and therefore may fail to detect certain abusive experiences (eg, those involving female perpetrators), which can have harmful health consequences if they remain unrecognized.

**Objective:**

The objective of this study is to overcome existing limitations in the literature by drawing on perspectives of black and Latino MSM and men who have sex with men and women (MSMW), as well as relevant service providers to better understand the role of, and the need to include, sexual abuse histories (eg, CSA) in treatment and counseling settings, with the long-term goal of improving assessment and health outcomes.

**Methods:**

We will conduct mixed-methods interviews, framed by an intersectionality approach, with 80 black and Latino men (40 MSM and 40 MSMW) in New York City (NYC), exploring appraisals of their formative sexual experiences, including those described as consensual but meeting criteria for CSA. We will also interview 30 local service providers representing substance abuse treatment, mental health care, and HIV prevention and outreach.

**Results:**

The study was launched in May 2017.

**Conclusions:**

This formative research will inform testable approaches to assessing and incorporating sexual abuse history into substance abuse treatment and other health and mental health services used by men with such histories.

## Introduction

### Background

Childhood sexual abuse (CSA) is nearly five times greater among men who have sex with men (MSM) than in the general male population [1], with prevalence as high as 27% according to findings from a meta-analysis [2]. Yet, society has scarcely recognized sexual abuse among boys outside of institutional settings [3-5]. MSM with CSA history are more likely to be black or Latino [6-10], or less likely to be white [11]. Although men who engage in same-sex encounters and have a history of CSA tend to be behaviorally bisexual or less likely to identify as gay [9-11], it is unclear whether and how the formative sexual experiences of men who have sex with men and women (MSMW) differ from those of MSM only. CSA is associated with adverse consequences among MSM and MSMW including substance use [2,8,9,12-14], poor mental health [8,9,11,13-19], high-risk sexual behavior [11,13,14,19-23], and revictimization [11,15,24]. Yet, investigating these histories is often confounded by underreporting [22,25-27] and varied definitions of abuse [2,27-31]. Moreover, large scale quantitative studies that confront participants with questions about unwanted or coercive sexual encounters during childhood are not likely to account for experiences perceived as consensual or normative that would otherwise meet criteria for abuse—that is, unrecognized by the victim [27,32-34]. Recognizing men’s own appraisals and interpretations of their childhood sexual experiences (hereafter referred to as CSE) may lead to a better understanding and assessment of abuse and its consequences [12,32,35-37].

Appraisals of CSE (eg, desired, unwanted, coercive, abusive) are influenced by social environmental factors including gender and culture. Men may be less likely than women to define certain CSE as abusive because their socialization into masculine gender roles leads them to fear that they will be perceived as homosexual or as victims [25,38,39]. Black and Latino MSM and MSMW may be under additional pressure to deny unwanted CSE as abusive or to appraise abusive experiences as consensual, including those with female perpetrators, as black and Latino cultures stress the most traditional forms of masculinity [29,32,40,41]. Indeed, these cultural forces may also pressure black and Latino men to conceal same-sex behavior [29,42-45] and to not identify themselves as gay or homosexual [29,46-48]. For these men, CSE with an older male and/or same-sex behavior may be sources of shame and social isolation [44,46,47,49-51]. Furthermore, research suggests that men with histories of sexual abuse are less comfortable with their same-sex attraction than men without such histories [13], and several studies have found that some black and Latino men who were sexually abused as children experience sexual identity confusion [41,52] or establish a link between their abusive experiences and current same-sex desire [32,50,52].

This study recognizes female perpetrators in the early sexual experiences of MSM and MSMW. To our knowledge, this is the first study to investigate formative sexual experiences of black and Latino MSM/W involving older female partners relative to their sexual identity development, adult sexual relationships, masculine and cultural norms, and psychosocial health outcomes. The majority of CSA research with men, specifically gay and bisexual men, would suggest that perpetrators are typically male. Female perpetrators are rarely accounted for in either research designs or analyses. Indeed, several studies have failed to report the gender or sex of perpetrators [8,12,17,21,29,33,53]. Others have omitted female perpetrators from their analysis and discussion [11,20,22,23]. In most of these studies, participants were asked to report incidents they perceived as abusive. If some men normalize early sexual experiences with older female partners, including those where conditions were coercive or there was an obvious age difference between the child/adolescent and partner, they are less likely to appraise—let alone report—such experiences as abusive [27]. Thus these studies will fail to detect situations of abuse that may have affected men’s sexual identity development, mental health, substance abuse, and adult sexual relationships.

However, there are a few exceptions [9,32,35]. In a recent study of MSM with a history of CSA, 42% reported at least one incident of abuse by an older female [9]. Another study found that 20 cases (out of 43) involved a female perpetrator, out of which only 4 men initially defined their experiences as abusive [35]. By recruiting and interviewing adult men who experienced their first sexual encounter(s) as a child or adolescent (with an older male, female, or both), the proposed study will overcome this limitation of prior research by increasing knowledge about female perpetrators, particularly, the way men consider these experiences as being influential to their sexual development, psychological and emotional functioning, and current risk-taking behaviors. Moreover, it responds to recent calls for research with black and Latino MSM/W to explore differences in appraisal and meanings of sexual abuse by perpetrator gender [29,52].

There is a need for qualitative research on CSE of black and Latino MSM/W. An in-depth qualitative exploration can yield a better understanding of the meanings that individuals assign to CSE [32,33] and the impact they believe those experiences have on their lives, particularly later substance use, mental health, and sexual behavior. As discussed above, much of the research on sexual abuse among MSM has been quantitative. Moreover, although several of these studies included sizable samples of black and Latino men [9,20,21,29,35,54], most included small percentages of MSMW.

Few studies have explored CSE with MSMW beyond quantitative assessment [10,32,37,50,52,55]. The findings from these qualitative studies are promising, suggesting that men perceive connections between abuse and their current same-sex behavior [32,37,50,52], including high-risk exploration of their sexuality [37], that abusive experiences may result in negative mental health consequences and coping strategies [10,32,50], and that being coerced into same-sex encounters by older men led to intense internal debate about their sexual identity and coming to terms with the combined pain and pleasure they derived from these experiences [55]. Nevertheless, these studies are limited; they had small sample sizes (Ns=13-33), some of which focused exclusively on black men, the original study aims were broader than childhood sexual experiences, and/or only a few included in-depth interviews. Therefore, it is critical to further explore through in-depth interviews how black and Latino MSM and MSMW perceive connections between their formative sexual experiences, cultural attitudes and masculinity norms, same-sex and opposite-sex attractions, and adult health and risk behavior. This study will also be critical in addressing shame, internalized homophobia, and fear of provider insensitivity, all of which may impact the disclosure of CSE (and potential detection of CSA by competent providers) and same-sex behavior and can reduce men’s willingness to seek substance abuse treatment and other health services [56-59].

### Service Provider Perspectives

Service providers, including substance use counselors and HIV outreach workers, may not be adequately prepared to recognize or safely address CSA with male clients. Male survivors of CSA, often overlooked as health care consumers, have unique clinical and psychological needs that warrant critical scientific inquiry [60]. Knowing that CSA history among gay and bisexual men is associated with negative outcomes (eg, revictimization, substance use, mental health, high-risk sexual behavior), researchers have called for interventions to incorporate CSA history into health services used by these men [2,29,37,50,56,61]. Recent evidence suggests that doing so can achieve reductions in psychological symptoms and sexual risk taking [62]. Yet, there is reason to believe that black and Latino MSM/W may be reluctant to access health services and substance abuse treatment due to fears of being stigmatized, potential exposure of their same-sex behavior, general provider mistrust, and perceptions that providers lack training in sexuality [56,63,57,64]. Furthermore, service providers may not be equipped to recognize the need for safe trauma-informed care, particularly as it relates to sexual abuse [65-70]. Currently, the inclusion of CSA in treatment and health-related service settings is nonexistent or hindered by assessments that use language assuming an experience is appraised as abusive, inadequate provider training, or lack of provider preparedness [65-70] that potentially places clients at risk of retraumatization. Thus, we need to understand attitudes and expectations of the providers and their potential clients regarding the inclusion of sexual histories in treatment and other health care settings, and how this should be done. We also need to account for the perceived level of preparedness among providers to address issues of abuse and sexuality among racial and ethnic minority men.

### Study Objective and Aims

To our knowledge, very few studies focus specifically on understanding formative sexual histories, particularly those experiences occurring before the age of 16 years (ie, CSE), of black and Latino MSM and MSMW. We are conducting mixed-methods interviews with 80 black and Latino men (40 MSM and 40 MSMW) in New York City (NYC), exploring appraisals of their formative sexual experiences, including those described as consensual but meeting criteria for abuse (Multimedia Appendix 1). We are also interviewing 30 local service providers representing substance abuse treatment, mental health care, and HIV/sexually transmitted infection (STI) prevention and outreach.

To address the need for better recognition of CSA at multiple levels, we will do the following: (1) investigate appraisals of CSE among black and Latino MSM and MSMW and evaluate these experiences using established criteria for defining sexual abuse; (2) examine appraisals of CSE relative to sexual identity formation, adult sexual relationships and behaviors, concealment of same-sex behavior, alcohol and drug use, psychological and emotional functioning, and cultural ideologies of masculinity among black and Latino MSM and MSMW; and (3) examine perspectives of black and Latino MSM and MSMW and relevant service providers to understand the role of, and the need to include, sexual history in treatment and counseling settings and to determine service providers’ preparedness to address CSE in treatment and counseling settings.

## Methods

### Intersectionality Framework

This study employs an intersectionality framework to examine appraisal and interpretation of CSE. Multiple social identities (eg, race, ethnicity, gender, sexual orientation) “intersect at the micro level of individual experience to reflect interlocking systems of privilege and oppression at the macro level” [71-73]. This framework holds that no one social identity is more important than others, and is ideally suited to qualitative or mixed-methods research as it is not intended to predict behaviors, health, or mental processes. It was included as a conceptual perspective for advancing lesbian, gay, bisexual, and transgender (LGBT) research in the Institute of Medicine Committee on LGBT Health report [74].

Through an intersectionality lens, and relying on an indirect approach to assessing CSE, this study will use mixed methods to consider how current appraisals of first sexual experiences are shaped by intersecting social identities (Multimedia Appendix 2). These include gender and perceptions of masculinity, black or Hispanic racial or ethnic identification, level of cultural estrangement, and sexual orientation. Measuring cultural estrangement and masculinity norms will help to specify racial and ethnic identity factors that may have particular salience for men’s appraisals of their CSE. Examining these factors using mixed methods permits data triangulation, enabling us to assess their reliability and validity. We further conceptualize that one’s appraisal of a childhood sexual encounter (or multiple encounters), together with these social identities, influences adult sexual relationships (including disclosure or concealment of same-sex behaviors), current and past use of alcohol or drugs, current psychological distress and emotional functioning, and whether or not an individual perceives a need for (or has attempted to access) substance use, mental health, or other health services. As has been suggested [75], attention will be paid to within- and between-group differences and similarities.

### Study Overview

This cross-sectional study will include in-person interviews with 80 black and Latino men (40 MSM and 40 MSMW) with a history of CSE, and interviews with 30 service providers about their preparedness to address issues of abuse with clients.

#### Interviews With Black and Latino MSM and MSMW in New York City (NYC)

##### Inclusion Criteria

Participants of any HIV status must: (a) be biologically male and identify himself as male; (b) be 18-50 years of age; (c) be black non-Hispanic or Hispanic/Latino; (d) have had sex with a man within the past 12 months; (e) have had at least one sexual experience before age 16 with a man or woman who was at least 18 years of age at the time; (f) be fluent in English; and (g) live in the NYC area.

##### Sampling Quotas

The study team will recruit a minimum of 30 men (38%) with a history of CSE with older female partners. This quota falls between estimates from our work with black MSMW (24% CSA by a female partner) [32] and those reported in 2 prior studies (CSA range 42%-47%) [9,35]. Furthermore, we will recruit 40 participants (20 black, 20 Latino) who have had sex with a woman (ie, MSMW) within the past 12 months. Finally, we will oversample by 5 to allow for incomplete interviews and/or unusable data.

##### Recruitment Strategy

We employ multiple recruitment methods (ie, internet advertising, community-based organizations) based on our experience in working with stigmatized populations [49,76-78]. Recruitment materials indicate that we are conducting a study on the sexual history of black and Latino men and their relationships with men and women, and that participants are compensated. Interested men are directed to the study website for more information and to be screened for eligibility. The research team has successfully used these methods in past research with MSM and MSMW [49,76,79-81].

###### Internet Advertising

The research team is utilizing social (eg, Facebook) and sexual networking websites to recruit participants who may or may not openly identify as having sex with men. Advertisements target users in our study population. Facebook has been used to recruit MSM [80,82] and young adults with a history of child maltreatment [83] for internet surveys. The research team purchased an email blast targeting local users of a sexual networking website for black and Hispanic MSM. We also intend to advertise through mental health services websites that provide support to men with a history of unwanted or abusive sexual experiences in childhood.

###### Community-Based Organizations

We are using relationships with local organizations to generate participant referrals [49] by posting or distributing recruitment materials and providing in-service presentations to staff members and clients. These organizations provide diverse services such as substance abuse, counseling and treatment, mental health services, medical care, and HIV/STI testing.

##### Screening Procedures

We screen for eligibility via a brief internet and mobile survey (Table 1). The study team has conducted numerous internet and mobile surveys in past research with MSM [79,80,84,85] and substance-using populations [86,87]. Eligible men interested in participating are asked to provide contact information (ie, email, phone) so that study staff can schedule an in-person interview.

##### Interview Meeting

After providing informed consent, participants complete a short interviewer-administered questionnaire to verify information collected during screening (ie, history of CSE, sociodemographics). Participants also complete a short series of survey measures to assess sexual orientation, concealment of same-sex behavior, HIV and STI testing history, substance use, psychological distress, suicidality, post-traumatic stress symptoms, cultural estrangement, masculinity, and history of intimate partner violence (estimated time is 25 min), see Table 1). The remainder of the meeting (estimated time is 1 h 35 min) is used to conduct the audio-recorded qualitative interview. Participants receive US $50 in compensation plus reimbursement for transportation costs.

###### Qualitative Interviews

We conduct semi-structured (focused) interviews [100] using a nondirective approach, whereby the interview trajectory is largely determined by participants. This provides them with an opportunity to explore their formative sexual experiences relative to matters of personal significance with the interviewer remaining attentive to issues of sexual identity and orientation, adult sexual relationships, identification with masculinity norms, substance use, emotional functioning, and concealment of same-sex behavior. We use an interview guide (Multimedia Appendix 3) that reflects a preliminary framework of factors expected to be important in understanding appraisal of CSE. As data are collected and the Principal Investigators (PIs) conduct initial analyses, elements of the guide may require revision and any important issues that emerge will be added. Interviewer training focused on skills for putting participants at ease, remaining neutral, establishing rapport, and eliciting rich data using an interview guide. Throughout data collection, the PIs will review randomly selected interviews to monitor their quality. The PIs also conduct regular debriefing sessions with the interviewers to review field notes and discuss concerns or unanticipated issues. This gives interviewers an opportunity for catharsis and allows for a preliminary examination of study aims within the context of each interview. Regular interaction of the PIs with the data and with field staff also serves as a verification step to ensure reliability and validity of the data [101].

###### Indirect Approach to Assessing Childhood Sexual Experiences

Researchers have called for more in-depth exploration of CSE among MSM and MSMW [29,32,37,50], with particular emphasis on how men perceive these experiences (eg, unwanted, consensual) [32]. Knowing that cognitive appraisal and interpretation of sexual abuse is associated with adjustment and coping strategies [29,39,102,103], it is critical that men be able to reflect on their experiences in their own terms. A major strength of this study is that it will differentiate experiences that men appraise as being abusive from those appraised as being consensual but meeting criteria for sexual abuse. We know from past research that this process allows for emerging connections among CSE, adult sexual relationships, and substance use behavior that may not be revealed in a quantitative assessment reliant on preimposed CSA definitions [32].

This is not to deny the value in definitions of CSA generated by quantitative research. On the contrary, a framework of agreed-upon variables to reinterpret men’s narratives of their experiences enables investigators to detect discrepancies that can be further addressed.

**Table 1:** Screening and interview measures (M).

M #	Phase	Measure
1a-1j	S^a^	Sociodemographics: (1a) sex at birth, (1b) gender identity and expression, (1c) age, (1d) ethnicity (Hispanic or non-Hispanic), (1e) race, (1f) income, (1g) education, (1h) religion and spirituality, (1i) housing, (1j) reside in NYC
2	I^b^	Klein Sexual Orientation Grid [88]: 21 items to assess past, present, and ideal sexual attraction, behavior, and fantasies; emotional, social, and lifestyle preferences; and self-identification
3a,b	S	Sexual partners: (3a) relationship status, (3b) gender of sexual partners in the past 12 months
3c	I	Sexual behavior in the past 6 months
4	I	Concealment of same-sex behavior: 7-item modified version [76] of the Self-concealment Scale [89]
5	I	HIV and sexually transmitted infection testing history: ever tested, when was the most recent test, results of most recent test
6a-6d	S, I	Childhood sexual experiences [32]: (6a) age at first sexual experience with a female (if applicable), (6b) age at first sexual experience with a male, (6c) age of female partner, (6d) age of male partner
7	I	Recent substance use [90,91]: 21 items to assess recent alcohol use, drug use, and related problems
8	I	Addiction Severity Index lite version [92]: Two items to assess substance use history
9	I	Mental Health Inventory[93]: 18-item measure to assess symptoms of psychological distress
10	I	Suicidality [94]: Single item to assess if participant ever seriously considered or tried to commit suicide
11	I	Post-traumatic stress symptoms [95]: 6-item screening tool
12	I	Cultural estrangement [96]: 4-item scale to assess perceived differences between one’s ideas or opinions and those of individuals in one’s primary and secondary groups
13	I	Male Role Norms Inventory-Short Form [97]: 21-item measure of traditional masculinity ideology
14	I	Intimate partner violence (IPV) [15]: three items to assess past year IPV and perpetrator
15	I	Resilience [98,99]: 2-item modified version of resilience
16	S	Provider interactions: Six items to assess perceptions of service in health care settings

^a^S implies administered as part of study screening.

^b^I implies administered as part of study interview.

Our approach: (1) allows men to discuss formative sexual experiences occurring before age 16 without imposing terminology that is commonly associated with abuse; (2) allows men to discuss why they appraise these experiences as abusive or consensual; and (3) allows researchers to systematically (re)appraise certain experiences as abusive based on details provided by the participant (ie, age differential, evidence of coercion, penetration) without influencing the narrative [32,34]. This strategy can be particularly useful for providers working with stigmatized populations, including black and Latino MSM/W who may be motivated by normative heterocentric pressures from within their respective communities to conceal abusive same-sex experiences. Indirect questioning has been used to elicit more accurate responses in screening for substance abuse [104], HIV risk [105], and intimate partner violence [106].

#### Interviews With Service Providers

For Aim 3, we are interviewing 30 NYC-based service providers (concurrently with the MSM and MSMW interviews) recruited through provider training networks and local organizations that specialize in substance abuse treatment, HIV prevention and counseling services, and mental health and other health-related services. Interviews are audio-recorded and take approximately 1.5 hours; providers receive $30 compensation. We begin with a few structured questions (eg, time in the profession, educational background, types of services offered to MSM and MSMW, courses and training in sexual education and crisis counseling); estimated time is 15 min. The remainder of the meeting focuses on perceived preparedness to incorporate sexual history and issues of sexuality into treatment or counseling and issues of trauma-informed care, including whether and how providers screen for CSA and what they do with this information as they learn it from clients. We present vignettes (of hypothetical clients) derived from previous research [32] to ascertain whether and how providers recognize potentially abusive sexual experiences. Follow-up (probing) questions ask how the provider would proceed with each client presented in the vignettes. We have also modified several topics from the interview guide used with black and Latino MSM/W (IG12-IG15) to examine provider perspectives on the need to incorporate sexual history (and how) in treatment and other counseling settings.

### Data Analysis and Interpretation

#### Qualitative Analysis

Interviews with black and Latino MSM/W will be transcribed before coding and thematic analysis [107,108]. The investigators will extract and examine interview text as it relates to the study aims. Using qualitative data analysis software (ATLAS.ti v. 8.0), the study team will be able to search for and extract text by combining codes: for example, “Appraisal of CSE with Male” AND “Concealment of Same-Sex Behavior.” In this example, retrieved segments of text will show only those interview sections where men discuss their appraisal of CSE with an older male partner and whether/how they conceal same-sex encounters. This process will be replicated throughout the analysis to examine each of the aims. The investigators will evaluate participant appraisals [32] of their CSE using established criteria for sexual abuse (*Aim 1*) [12,35,50]. The following will be coded for each of the narratives and validated with study measures: participant’s age at the time of his experience(s); age of sexual partner(s); and the nature of the relationship or encounter (eg, power differential, evidence of coercion). Whenever possible, we will consult information regarding age of consent laws to further determine whether descriptions of sexual experiences are consistent with those of abuse. Furthermore, this process will allow us to consider how the intersection of social identities may influence appraisals of CSE (*Aim 2*)—for example, current relationships with women (IG8), identification with masculinity norms (IG7), and CSE with an older female (IG3).

#### Analysis of Service Provider Interviews (Aim 3)

Interviews with service providers will be transcribed and coded before analysis. We will analyze and integrate provider data in several ways. First, using data from both providers and MSM/W, we will compare perceptions of the need to incorporate sexual history into treatment and counseling settings. We will also examine acceptable intervention formats with sexual abuse issues from the perspectives of potential clients and providers (IG13-IG15). Second, provider responses to vignettes will reveal patterns in perceived abuse and preliminary findings as to the acceptability of using an indirect approach to assessing CSE (and ultimately, abuse). Third, using provider accounts of their experiences with client sexual histories, in conjunction with how they respond to the vignettes, we will derive a preliminary assessment of the level of preparedness to address issues of abuse and sexuality in diverse treatment and counseling settings.

#### Quantitative Analysis (Aims 1 and 2)

The study aims will be primarily addressed through qualitative analyses. However, the proposed sample (N=80 black and Latino MSM and MSMW) will allow for some supplemental quantitative analyses [109]. Validity of self-reported data will be assessed by comparing eligibility survey information, survey measures administered during the interview meeting (see Table 1), and the qualitative interview [110]. For statistical analyses, we will use the conventional alpha level (.05). For Aims 1 and 2, we will run all frequencies to describe sample characteristics (eg, age, sexual orientation, HIV status) and conduct chi-square or analysis of variance tests to compare key characteristics (eg, differences [racial/ethnic, sexual orientation, concealment of same-sex behavior, perpetrator gender, and perpetrator age] between men who perceive their experiences as abusive versus those who do not). We will also examine potential differences between those participants who described consensual experiences that met conditions for CSA and those who reported abusive experiences.

Appraisals of CSE are a critical outcome of the proposed study. Power calculations for this outcome (example below) are based on estimates from our previous study with nongay-identified MSMW [32]. In that study, 27.5% of men reporting a sexual experience during childhood perceived it as abusive. Estimated power for a two-sample paired proportions test is based on the following: proportion of experiences appraised as abusive by the participant and proportion of experiences (re)appraised as abusive by the study investigators. Assuming an alpha of .05 and a conservative change (increase) of 30% in abuse appraisals [32], the proposed sample sizes (N=80; n=40 per subgroup [MSM, MSMW; black, Latino]) have sufficient power (>.90) to detect a difference.

### Data Integration

We will employ “Best Practices” for mixed methods research [111] to integrate quantitative and qualitative data. Quantitative data will be used to identify subgroups to compare emergent qualitative themes. For example, cases of problematic substance use will be identified before reviewing the qualitative data pertaining to IG9 (coping with CSE) and IG10 (alcohol or drug initiation). Quantitative and qualitative data will be used to assess multiple social identities expected to influence appraisal (Multimedia Appendix 2). Thus, we will be able to examine, for example, how black MSM who identify themselves as gay understand their CSE with an older male compared with heterosexually-identified black MSMW who actively conceal same-sex behavior. We will also analyze potential cultural similarities in CSE appraisal and interpretation—for example, Latino and black MSMW with low levels of cultural estrangement and high identification with traditional masculine norms. Attention will be paid to similarity and diversity within and between groups throughout the analysis [75]. Finally, employing both qualitative and quantitative analyses can enhance the validity of study findings [110,111].

## Results

The study received Institutional Review Board approval and was launched in May 2017. Data collection is underway and results are forthcoming.

## Discussion

### Limitations

We recognize that this formative study will not have sufficient resources to recruit a truly representative sample. For example, nonspeakers of English will not be included. Moreover, recruitment methods favor those with internet access, but this may be somewhat offset by recruitment from community-based organizations. Much of the data will be retrospective and despite measures that we have incorporated to minimize recall bias, the possibility remains that some accounts may suffer from memory suppression.

### Practical Significance

Findings will inform an R01 proposal to develop and test an alternative CSA instrument accompanied by a training curriculum for use in health services and research settings. Specifically, data from black and Latino MSM/W will be translated into a preliminary set of testable questions to indirectly detect sexual abuse perpetrated against male participants or clients. Study findings from service providers will offer insight into how qualitative assessments of sexual history can be used in conjunction with substance use measures and mental health screenings to better understand the impact of abuse; inform strategies to improve linkage to care; elucidate the degree of acceptability, preparedness, and potential discomfort among service providers in taking childhood sexual histories; and inform training in the use of this alternative instrument.

## Appendix

Multimedia Appendix 1 NIH summary statement (peer-review report).

Multimedia Appendix 2 Intersecting social identities and their influence on CSE appraisal and interpretation.

Multimedia Appendix 3 Qualitative interview guide topics.

## Abbreviations

ASI: Addiction Severity Index
CSA: childhood sexual abuse
CSE: childhood sexual experience
IPV: intimate partner violence
LGBT: lesbian, gay, bisexual, and transgender
MSM: men who have sex with men
MSMW: men who have sex with men and women
NYC: New York City
STI: sexually transmitted infection
