# Supplementary material for: Understanding the Impact of Childhood Sexual Abuse on Men’s Risk Behavior: Protocol for a Mixed-Methods Study
Source: JMIR Res Protoc. 2018 Feb 26;7(2):e62. doi: 10.2196/resprot.9071 (PMC5847822; doi:10.2196/resprot.9071)
Supplement: Multimedia Appendix 1 [file resprot_v7i2e62_app1.pdf]

SUMMARY STATEMENT

**PROGRAM CONTACT:**	**(Privileged Communication)**	***Release Date:***	**02/23/2016 10:13 AM**
		Revised Date:	

Application Number: **1 R21 HD084939-01A1**

Principal Investigators (Listed Alphabetically):

BENOIT, ELLEN (Contact)
DOWNING, MARTIN

Applicant Organization: NATIONAL DEVELOPMENT & RES INSTITUTES

Review Group: **HDEP**
Health Disparities and Equity Promotion Study Section

Meeting Date:	**02/04/2016**	***RFA/PA:***	**PA15-263**
Council:	**MAY 2016**	***PCC:***	**PDB -SN**
Requested Start:	**07/01/2016**	***Dual PCC:***	**CM/DJJ**
		Dual IC(s):	**DA, MD, MH**

Project Title: **Abuse Appraisals among Black and Latino Men**

SRG Action: **Impact Score:17 Percentile:4 +**
Next Steps: **Visit http://grants.nih.gov/grants/next_steps.htm**
Human Subjects: 30-Human subjects involved - Certified, no SRG concerns
Animal Subjects: 10-No live vertebrate animals involved for competing appl.
Gender: 1A-Both genders, scientifically acceptable
Minority: 1A-Minorities and non-minorities, scientifically acceptable
Children: 1A-Both Children and Adults, scientifically acceptable
Clinical Research - not NIH-defined Phase III Trial

Project Year	Direct Costs Requested	Estimated Total Cost
1	150,000	228,021
2	125,000	190,018
TOTAL	275,000	418,039

ADMINISTRATIVE BUDGET NOTE: The budget shown is the requested budget and has not been adjusted to reflect any recommendations made by reviewers. If an award is planned, the costs will be calculated by Institute grants management staff based on the recommendations outlined below in the COMMITTEE BUDGET RECOMMENDATIONS section.

1R21HD084939-01A1 Benoit, Ellen

RESUME AND SUMMARY OF DISCUSSION: This project will investigate appraisals of formative childhood sexual experiences among Black and Latino sexual minority men who have a history of childhood sexual abuse (CSA) and will examine provider perspectives regarding the inclusion of these experiences into treatment and counseling settings. The proposed study addresses a significant and understudied public health issue and if successful, findings will likely advance our understanding of how CSA experiences of sexual minority men influence their HIV/AIDS risk prevention and treatment needs and inform strategies to reduce the negative health consequences of CSA among these at risk populations. The resubmission is strengthened by changes that are highly responsive to concerns identified in the previous review. During the discussion, the panel noted several strengths of the application; including, a strong mixed-method approach, use of an intersectionality framework and an innovative consideration of female perpetrators. Plans to differentiate experiences that men appraise as abusive from those appraised as consensual but meeting criteria for sexual abuse was an additional notable strength. Overall, the proposed research is viewed as being outstanding, with potentially highly significant outcomes that could have a major impact in the field of sexual minority health.

DESCRIPTION (provided by applicant): Black and Latino sexual minority men are likely to have higher rates of childhood sexual abuse (CSA) than their White counterparts. CSA history among sexual minority men is associated with adverse consequences including substance misuse, poor mental health, re-victimization, and high-risk sexual behavior. Because Black and Latino cultures stress traditional forms of masculinity, men may be under pressure to deny certain childhood sexual experiences as abusive out of fear of being perceived as gay or as victims. Most CSA research with sexual minority men relies on quantitative measures that often preclude consideration of cultural variations in how formative sexual experiences are understood, and are based on inconsistent and/or overly restrictive definitions of abuse. Moreover, limited assessment methods may result in failure to detect some abusive experiences (e.g., those involving female perpetrators), which can have harmful health consequences if they remain unrecognized. These issues indicate a need to broaden the conceptualization of CSA to recognize men's appraisals and interpretations of their childhood sexual experiences (CSE). To that end, we will conduct mixed-methods interviews regarding CSE with 40 Black and 40 Latino sexual minority men in New York City. Researchers are calling for interventions that incorporate CSA history into substance abuse treatment and other health services used by men who have sex with men (MSM) and men who have sex with men and women (MSMW). However, these men may be reluctant to access such services for fear of stigma, exposure of their same-sex behavior, general provider mistrust, and perceptions that providers lack training in sexuality. Further, service providers may not be equipped to recognize the need for safe trauma-informed care, particularly as it relates to sexual abuse. To understand provider experiences and treatment protocols, we will interview 30 service providers regarding whether and how sexual histories are addressed in treatment and other health care settings. We propose the following specific aims: 1) to investigate appraisals of childhood sexual experiences among Black and Latino MSM and MSMW and to evaluate these experiences using established criteria for defining sexual abuse; 2) to examine appraisals of childhood sexual experiences relative to sexual identify formation, adult sexual relationships and behaviors, concealment of same-sex behavior, alcohol and drug use, psychological and emotional functioning, and cultural ideologies of masculinity among Black and Latino MSM and MSMW; 3) to examine perspectives of Black and Latino MSM and MSMW and relevant service providers to: a) understand the role of, and need to include, sexual history in treatment and counseling settings; and b) determine service providers' preparedness to address childhood sexual experiences in treatment and counseling settings. This formative research will inform testable approaches to assessing and incorporating sexual abuse history into substance abuse treatment and other health and mental health services used by men with such histories.

PUBLIC HEALTH RELEVANCE: Statement Childhood sexual abuse (CSA) remains a critical public health issue among Black and Latino sexual minority men, as it is associated with multiple negative outcomes including substance misuse, poor mental health, re-victimization, and high-risk sexual behavior. Most CSA research with sexual minority men relies on quantitative assessment that often precludes consideration of cultural variations in how formative sexual experiences are understood, are based on inconsistent or overly restrictive definitions of abuse, and therefore may fail to detect certain abusive experiences (e.g., those involving female perpetrators), which can have harmful health consequences if they remain unrecognized. This mixed-methods study will overcome existing limitations by drawing on perspectives of Black and Latino sexual minority men and relevant service providers to better understand the role of, and need to include, sexual abuse histories in treatment and counseling settings, with the long-term goal of improving assessment and health outcomes.

CRITIQUE 1

Significance: 1
Investigator(s): 1
Innovation: 2
Approach: 2
Environment: 1

Overall Impact: This application is likely to be impactful for improving the health of Black and Latino MSM/W. Childhood sexual abuse (CSA) is a critical issue disproportionally affecting Black and Latino sexual minority men. As the application stated "CSA research with sexual minority men relies on quantitative assessment that often precludes consideration of cultural variations in how formative sexual experiences are understood, are based on inconsistent or overly restrictive definitions of abuse, and therefore may fail to detect certain abusive experiences (e.g., those involving female perpetrators), which can have harmful health consequences if they remain unrecognized." The findings from this application are likely to overcome existing limitations in CSA research.

1. Significance:
Strengths

- Childhood sexual abuse (CSA) is nearly five times greater among men who have sex with men (MSM) than the general male population with prevalence as high as 27% according to a recent meta-analysis.
- Appraisals of childhood sexual experiences (e.g., desired, unwanted, coercive and abusive) are influenced by social environmental factors including gender and culture.
- Men may be less likely than women to define certain childhood sexual experiences as abusive because their socialization into masculine gender roles leads them to fear that they will be perceived as homosexual or as victims.
- Assessments of CSA among sexual minority men have been problematic.
- Providers may not be adequately prepared to recognize or safely address CSA with male clients.

Weaknesses

- None noted.

2. Investigator(s):
Strengths

- Excellent.
- Dr. Benoit (PI) is a qualitative sociologist with substantial experience conducting and managing research on substance use and related health risk behavior among low-income African American adults in New York City. She was the PI on a qualitative study with substance-using non-gay-identified Black men who have sex with men and women (MSMW) (R03 DA024997).

- Dr. Downing (PI) has a PhD in psychology and is a mixed-methods researcher with extensive experience in project management. He has worked with sexual minority and gender variant populations.
- [redacted] and [redacted] are leading scholars on sexual-mental health of sexual minorities.

Weaknesses

- None noted.

3. Innovation:
Strengths

- There is a need for qualitative research on CSE of Black and Latino sexual minority men.
- Female perpetrators in the early sexual experiences of sexual minority men are recognized in this application.
- A major strength of this proposed study is that it will differentiate experiences that men appraise as abusive from those appraised as consensual but meeting criteria for sexual abuse.
- This study will employ an intersectionality framework to examine appraisal and interpretation of childhood sexual experiences.

Weaknesses

- None noted.

4. Approach:
Strengths

- This application is based on two preliminary studies which findings documented consensual CSA between Black MSM and specific behavioral risk consequences attributable to CSA experience.
- The methodological approach is appropriate for the proposed specific aims: Methods include: In-person interviews will be conducted with 40 Black and 40 Latino MSM/W with CSE history and with 30 service providers about their preparedness to address issues of abuse with clients.
- The inclusion of cultural estrangement in the conceptual framework and measurements is a major strength of the proposed application.
- The use of provider responses to vignettes to examine the acceptability of using an indirect approach to assessing CSE (and ultimately, abuse).
- Practical significance section is a major strength of this application.

Weaknesses

- The following weakness are minor and do not reduce enthusiasm in this application and its strengths outweigh significantly the identified minor weaknesses.
- MINOR: Since the justification for this study is the examination of the influences of gender and culture, this study would benefit from cultural issues more explicitly in the qualitative and quantitative measures, this is particularly important since non-English speakers are excluded. Suggestion: Include measures of acculturation and assimilation as applicable to Latinos and non-US born Blacks.
- MINOR: The qualitative data analysis of the interviews with Black and Latino MSM/MSMW presents only the management of the narrative data but does not provide insights into how the research questions will be answered. In fact the illustration presented: “The investigators will use Atlas.ti to extract and examine interview text as it relates to the study aims. Atlas.ti also allows users to search for and extract text by combining codes: e.g., “Appraisal of CSE with Male” AND Concealment of Same-Sex Behavior’ does not require Atlas.ti since the researchers are doing a quantitative survey at the start of the interview with a series of psychometric measures see Table 1, measure #4 (Concealment of same-sex behavior: 7-item modified

version96 of the Self-Concealment) that can provide a more effective way of segregating the qualitative data for analysis.
- MINOR: The research questions guiding the secondary data analysis are not clear. As well, it is not clear how the effect of the proposed quota sampling framework on the quantitative analysis is considered.
- MINOR: The only masculinity quantitative measure is from Hammond, et al., 2010. Has the scale been validated with MSM/W and/or with Latinos?

5. Environment:
Strengths
- NDRI and PHS are excellent research environments conducive for the type of research proposed.

Weaknesses
- None noted.

Protections for Human Subjects:
Acceptable Risks and/or Adequate Protections
- Acceptable.

Data and Safety Monitoring Plan (Applicable for Clinical Trials Only):
Not Applicable (No Clinical Trials)

Inclusion of Women, Minorities and Children:
- Sex/Gender: Distribution justified scientifically
- Race/Ethnicity: Distribution justified scientifically
- Inclusion/Exclusion of Children under 21: Including ages < 21 justified scientifically
- Acceptable.

Vertebrate Animals:
Not Applicable (No Vertebrate Animals)

Biohazards:
Not Applicable (No Biohazards)

Resubmission:
- The proposed application is highly responsive to prior reviews.

Budget and Period of Support:
Recommend as Requested
- No concerns identified.

CRITIQUE 2

Significance: 1
Investigator(s): 1
Innovation: 1
Approach: 2
Environment: 1

Overall Impact: Using a mixed-method methodology, this study aims to elucidate childhood sexual abuse (CAS) among a sample of Black and Latino men who have sex with men (MSM). These men

have higher prevalence of CAS than their white counterparts. Individuals with CAS have higher rate of substance abuse that those who do not. The study has high public health significance and if successful, findings would inform substance abuse treatment and other mental health services targeting these men. The inclusion of female perpetrators is an innovation.

1. Significance:
Strengths
- MSM (especially Black and Latino) have a higher prevalence of CAS than their white counterparts.
- Those with CAS have higher rate of substance abuse that those who do not.
- Use of an intersectionality framework (e.g., race/ethnicity, sexual orientation).

Weaknesses
- None noted.

2. Investigator(s):
Strengths
- This is an impressive team with both content expertise and experience working with the target population.

Weaknesses
- None noted.

3. Innovation:
Strengths
- The inclusion of female perpetrator is an innovation.

Weaknesses
- None noted.

4. Approach:
Strengths
- Well-articulated study design.
- Perspectives regarding CAS from both MSM and health care providers.
- Inclusion of a licensed therapist.

Weaknesses
- What is the distinction between “non-directive approaches” from “indirect questioning?” The statement “…. allows researchers to systematically (re) appraise certain experiences as abusive or consensual” could be understood as “indirect” but not “non-direct.”

5. Environment:
Strengths
- Appropriate.

Weaknesses
- None noted.

Protections for Human Subjects:
Acceptable Risks and/or Adequate Protections
- No concerns identified.

Data and Safety Monitoring Plan (Applicable for Clinical Trials Only):
Not Applicable (No Clinical Trials)

Inclusion of Women, Minorities and Children:

- Sex/Gender: Distribution justified scientifically
- Race/Ethnicity: Distribution justified scientifically
- Inclusion/Exclusion of Children under 21: Including ages < 21 justified scientifically
- No concerns identified.

Vertebrate Animals:
Not Applicable (No Vertebrate Animals)

Biohazards:
Not Applicable (No Biohazards)

Resubmission:
- Overall, this amended application is responsive to previous critiques.

Budget and Period of Support:
Recommend as Requested
- No concerns identified.

CRITIQUE 3

Significance: 1
Investigator(s): 1
Innovation: 2
Approach: 2
Environment: 1

Overall Impact: The overarching goals of this study are to conduct qualitative interviews (and some quantitative assessments) with 80 black and Latino MSM and MSMW who have a history of CSA in order to understand the specific circumstances of their abuse experiences and how they have impacted their long term health behaviors. The overall merit and impact of this study is high based on the many strengths of the study: (1) The focus on a highly marginalized and stigmatized population based both on sexual orientation and CSA victimization; (2) The use of qualitative interviews to explore in depth the CSA experience and gain a better understanding of how the participant conceptualizes the abuse (e.g., as consensual non abuse vs. abuse); (3) Use of the data to develop a future questionnaire that better captures the nuanced experiences and different details of early sexual experiences in order to understand how CSA happens/unfolds among the survivors; (4) Understanding detailed characteristics of the perpetrator, particularly highlighting the often ignored role of female perpetrators; (5) Using a small data set to conduct some preliminary quantitative analyses to compare those who believe their early experiences were abusive versus those who do not in order to determine how that impacts potential health outcomes. The strengths of this study outweigh the few but necessary limitations of the proposal: (1) because it is an exploratory study the sample is small and generalizability is limited, but this is still an important first step in understanding the problem. (2) The data will be retrospective, however in order to conduct this kind of research, by default, it has to be retrospective whether studying teenagers or adults. Following a very large group of children with CSA experiences overtime, in order to have a large enough group of MSM from that cohort in order to examine longitudinal trajectories of health is problematic. The proposed study is a good alternative until the preliminary data justify a larger longitudinal and comprehensive study.

1. Significance:
Strengths

- This topic is of utmost importance to help understand risk for negative health outcomes among black and Latino MSM and MSMW.
- CSA in this population, while there are some studies among adult MSM in the HIV literature, has largely been ignored because of the stigma associated with the abuse. Conducting these in depth interviews with victims is sorely needed to fully understand the phenomenon and how to assess/prevent/intervene in the future.
- Using the intersectionality theory/model is apropos and an important way to conceptualize the combined risk due to two highly stigmatized identities/experiences.

Weaknesses

- Minor weakness is that without comparing black and Latino MSM to white MSM some of the opportunities to truly understand intersectionality (differences due to stigmatized identities and associated stressors) are lost.

2. Investigator(s):

Strengths

- The investigative team is very strong. The proposed PI Benoit has a very strong track record of peer-reviewed publications and NIH funding to study risk/resilience among black MSM, to conduct formative/qualitative research, and to study substance use and abuse including policy expertise.
- The proposed co-PI Downing, although an early stage investigators, has content/substantive expertise with bisexual men and a history of scholarly peer-reviewed work which focuses on risk among this population.
- The added expertise (in response to reviewer critiques) of [redacted] is appropriate. He brings CSA experience and as a consultant, can contribute to developing the interview instruments and with data analysis and interpretation.

Weaknesses

- None noted.

3. Innovation:

Strengths

- The focus on gaining an in depth understanding of CSA experiences among black and Latino MSM and MSMW is highly novel and imperative to the future of this research topic.

Weaknesses

- A minor comment is that some of the proposed data analyses are common methods that are appropriate but not very innovative.

4. Approach:

Strengths

- Mixed methods approach using qualitative interviews and some basic quantitative data analyses in order to understand CSA experiences is a strength.
- Focusing on childhood sexual experiences, and the indirect approaches to understanding the stigmatized topic of CSA is a strength.

Weaknesses

- A minor and necessary weakness is a small sample size and recruitment methods that may introduce some biases regarding generalizability of the findings but this is a necessary first step to justifying a larger scale study that can overcome these limitations.
- Virtually all CSA research with adolescents and beyond is retrospective. It is simply an unavoidable component of this line of research. The results from this study may prove fruitful and important enough to justify a large scale longitudinal study with children under age 16 with abuse experiences to minimize the complications associated with retrospective biases.

5. Environment:
Strengths

- The National Development and Research Institutes (established 1967) and the Institute for Special Populations Research is comprised of sound research, budgetary, and administrative infrastructures, and in total has 70 staff members and currently 65 research projects. The thorough description of the NDRI shows that it has ample resources to conduct the proposed research.

Weaknesses

- None noted.

Protections for Human Subjects:
Acceptable Risks and/or Adequate Protections

- Risks and protections were adequately addressed. Just as a suggestion, added protection could include verbal consent rather than signed consent in order to minimize chances of an unauthorized person viewing the signature on the consent form. Further, sending one home with a copy of the consent form could impose risks of breach in confidentiality. If the participant inadvertently leaves the consent form in a place where others can see it, and deduce that the individual is a participant, and then know about the individual's private information such as sexual orientation or CSA history.

Data and Safety Monitoring Plan (Applicable for Clinical Trials Only):
Not Applicable (No Clinical Trials)

Inclusion of Women, Minorities and Children:

- Sex/Gender: Distribution justified scientifically
- Race/Ethnicity: Distribution justified scientifically
- Inclusion/Exclusion of Children under 21: Including ages < 21 justified scientifically
- Children 18-20 will be included. Men only is justified given study of MSM. Black and Latino men are the study population.

Vertebrate Animals:
Not Applicable (No Vertebrate Animals)

Biohazards:
Not Applicable (No Biohazards)

Resubmission:

- The resubmission was very responsive to the previous review.

Budget and Period of Support:
Recommend as Requested

- No concerns identified.

THE FOLLOWING SECTIONS WERE PREPARED BY THE SCIENTIFIC REVIEW OFFICER TO SUMMARIZE THE OUTCOME OF DISCUSSIONS OF THE REVIEW COMMITTEE, OR REVIEWERS' WRITTEN CRITIQUES, ON THE FOLLOWING ISSUES:

PROTECTION OF HUMAN SUBJECTS (Resume): ACCEPTABLE

INCLUSION OF WOMEN PLAN (Resume): ACCEPTABLE

INCLUSION OF MINORITIES PLAN (Resume): ACCEPTABLE

INCLUSION OF CHILDREN PLAN (Resume): ACCEPTABLE

COMMITTEE BUDGET RECOMMENDATIONS: The budget was recommended as requested.

Footnotes for 1 R21 HD084939-01A1; PI Name: Benoit, Ellen

+ Derived from the range of percentile values calculated for the study section that reviewed this application.

NIH has modified its policy regarding the receipt of resubmissions (amended applications). See Guide Notice NOT-OD-14-074 at http://grants.nih.gov/grants/guide/notice-files/NOT-OD-14-074.html. The impact/priority score is calculated after discussion of an application by averaging the overall scores (1-9) given by all voting reviewers on the committee and multiplying by 10. The criterion scores are submitted prior to the meeting by the individual reviewers assigned to an application, and are not discussed specifically at the review meeting or calculated into the overall impact score. Some applications also receive a percentile ranking. For details on the review process, see http://grants.nih.gov/grants/peer_review_process.htm#scoring.

MEETING ROSTER
Health Disparities and Equity Promotion Study Section
Healthcare Delivery and Methodologies Integrated Review Group
CENTER FOR SCIENTIFIC REVIEW

HDEP
02/04/2016 - 02/05/2016

CHAIRPERSON(S)

SOMMERS, MARILYN S, PHD, RN
LILLIAN S BRUNNER PROFESSOR OF MEDICAL SURGICAL NURSING
SCHOOL OF NURSING
UNIVERSITY OF PENNSYLVANIA
PHILADELPHIA, PA 19104

MEMBERS

ANTHONY, RENAISA SPRING, MD *
DEPUTY DIRECTOR
CENTER FOR REDUCING HEALTH DISPARITIES
COLLEGE OF PUBLIC HEALTH
UNIVERSITY OF NEBRASKA MEDICAL CENTER
OMAHA, NE 68198

ARCOLEO, KIMBERLY JOAN, PHD *
ASSOCIATE PROFESSOR
ASSOCIATE DEAN FOR RESEARCH
AND TRANSDISHIPLINARY SCHOLARSHIP
COLLEGE OF NURSING
OHIO STATE UNIVERSITY
COLUMBIA, OH 43210

BERRY, CAROLYN ANNE, PHD
ASSOCIATE PROFESSOR
DEPARTMENT OF POPULATION HEALTH
NEW YORK UNIVERSITY SCHOOL OF MEDICINE
NEW YORK, NY 10016

BOUTIN-FOSTER, CARLA, MD *
ASSOCIATE PROFESSOR
DIVISION OF GENERAL INTERNAL MEDICINE
DEPARTMENT OF MEDICINE
WEILL CORNELL MEDICAL COLLEGE
CORNELL UNIVERSITY
NEW YORK, NY 10021

CABASSA, LEOPOLDO J, PHD *
ASSOCIATE PROFESSOR
COLUMBIA UNIVERSITY
SCHOOL OF SOCIAL WORK
NEW YORK, NY 10032

CLARK, DANIEL O, PHD *
ASSOCIATE PROFESSOR
DEPARTMENT OF MEDICINE
INDIANA UNIVERSITY
INDIANAPOLIS, IN 46202

ELDER, JOHN P, PHD
PROFESSOR OF PUBLIC HEALTH
GRADUATE SCHOOL OF PUBLIC HEALTH
SAN DIEGO STATE UNIVERSITY
SAN DIEGO, CA 92123

FREUND, KAREN, MD *
PROFESSOR AND VICE CHAIR OF MEDICINE
TUFTS MEDICAL CENTER
TUFTS UNIVERSITY
BOSTON, MA 02111

GARCIA, VICTOR Q, PHD *
DISTINGUISHED UNIVERSITY PROFESSOR AND DIRECTOR
MID-ATLANTIC RESEARCH AND
TRAINING INSTITUTE (MARTI)
INDIANA UNIVERSITY OF PENNSYLVANIA
INDIANA, PA 15701

GRAVLEE, CLARENCE C *
ASSOCIATE PROFESSOR
DEPARTMENT OF ANTHROPOLOGY
UNIVERSITY OF FLORIDA
GAINESVILLE, FL 32611

HALL, LYNNE ANDERSON, BSN, DRPH, RN *
PROFESSOR
SCHOOL OF NURSING
UNIVERSITY OF LOUISVILLE
LOUISVILLE, KY 40207

HOWARD, VIRGINIA J, PHD *
PROFESSOR
DEPARTMENT OF EPIDEMIOLOGY
SCHOOL OF PUBLIC HEALTH
UNIVERSITY OF ALABAMA AT BIRMINGHAM
BIRMINGHAM, AL 35294

KAVANAUGH-LYNCH, MARION HELEN ELOISE, MD *
DIRECTOR
CALIFORNIA BREAST CANCER RESEARCH PROGRAM
UNIVERSITY OF CALIFORNIA
OAKLAND, CA 94612

KLASSEN, ANN CARROLL, PHD
PROFESSOR, ASSOCIATE DEAN FOR RESEARCH
DEPARTMENT OF COMMUNITY HEALTH AND PREVENTION
SCHOOL OF PUBLIC HEALTH
DREXEL UNIVERSITY
PHILADELPHIA, PA 19104

MARSHAL, MICHAEL P, PHD *
ASSOCIATE PROFESSOR
DEPARTMENT OF PSYCHIATRY
SCHOOL OF MEDICINE
UNIVERSITY OF PITTSBURGH
PITTSBURGH, PA 15213

MATTHEWS, ALICIA K, PHD
PROFESSOR
DEPARTMENT OF HEALTH SYSTEMS SCIENCES
COLLEGE OF NURSING
UNIVERSITY OF ILLINOIS AT CHICAGO
CHICAGO, IL 60612

MENON, USHA, FAAN, PHD, RN
ASSOCIATE DEAN FOR RESEARCH AND GLOBAL ADVANCES
COLLEGE OF NURSING
UNIVERSITY OF ARIZONA
TUCSON, AZ 85721

MUNOZ-LABOY, MIGUEL A, DPH *
ASSOCIATE PROFESSOR
SCHOOL OF SOCIAL WORK
TEMPLE UNIVERSITY
PHILADELPHIA, PA 19122

NUNEZ-SMITH, MARCELLA, MD *
ASSOCIATE PROFESSOR OF MEDICINE AND PUBLIC HEALTH
EQUITY RESEARCH AND INNOVATION CENTER
YALE UNIVERSITY
NEW HAVEN, CT 06520

ORTEGA, ALEXANDER N, PHD
PROFESSOR
UCLA CENTER FOR POPULATION HEALTH
AND HEALTH DISPARITIES
SCHOOL OF PUBLIC HEALTH
UNIVERSITY OF CALIFORNIA
LOS ANGELES, CA 90095

POSTON, WALKER S, PHD *
DEPUTY DIRECTOR AND SENIOR PRINCIPAL INVESTIGATOR
INSTITUTE FOR BIOBEHAVIORAL HEALTH RESEARCH
CENTER FOR FIRE, RESCUE AND EMS HEALTH RESEARCH
NATIONAL DEVELOPMENT
AND RESEARCH INSTITUTES NDRI
NEW YORK, NY 10010

PREWITT, THERESA ELAINE, DRPH *
ASSOCIATE PROFESSOR
DEPARTMENT OF HEALTH POLICY AND MANAGEMENT
DEP. DIR ARKANSAS CENTER FOR HEALTH DISPARATIES
FAY W. BOOZMAN COLLEGE OF PUBLIC HEALTH
UNIVERSITY OF ARKANSAS FOR MEDICAL SCIENCES
LITTLE ROCK, AR 72205

RADECKI BREITKOPF, CARMEN, PHD *
ASSOCIATE PROFESSOR
DIVISION OF HEALTH CARE POLICY AND RESEARCH
DEPARTMENT OF HEALTH SCIENCES RESEARCH
MAYO CLINIC COLLEGE OF MEDICINE
ROCHESTER, MN 55905

REGENSTEIN, MARSHA J, PHD
PROFESSOR
DEPARTMENT OF HEALTH POLICY
GEORGE WASHINGTON UNIVERSITY
WASHINGTON, DC 20052

SHUMWAY, MARTHA, PHD *
ASSOCIATE PROFESSOR
DEPARTMENT OF PSYCHIATRY
UNIVERSITY OF CALIFORNIA SAN FRANCISCO
SAN FRANCISCO, CA 94143

STROUP, ANTOINETTE MARIE, PHD *
ASSOCIATE PROFESSOR
DEPARTMENT OF EPIDEMIOLOGY
DIRECTOR, NEW JERSEY STATE CANCER REGISTRY
RUTGERS, THE STATE UNIVERSITY OF NEW JERSEY
SCHL OF PUBLIC HLTH & CANCER INST OF NEW JERSEY
NEW BRUNSWICK, NJ 08903

TEHRANIFAR, PARISA, DPH *
ASSISTANT PROFESSOR
EPIDEMIOLOGY
MAILMAN SCHOOL OF PUBLIC HEALTH
COLUMBIA UNIVERSITY
NEW YORK, NY 10032

THOMAS, STEPHEN B, PHD
DIRECTOR, MARYLAND CENTER FOR HEALTH EQUITY
PROFESSOR, DEPARTMENT OF HEALTH SERVICES
ADMINISTRATION, SCHOOL OF PUBLIC HEALTH
UNIVERSITY OF MARYLAND, COLLEGE PARK
COLLEGE PARK, MD 20742

VIDRINE, JENNIFER IRVIN, PHD *
PEGGY AND CHARLES STEPHENSON ENDOWED CHAIR IN
CANCER DEPUTY DIRECTOR FOR TOBACCO RESEARCH
AND DIRECTOR, OKLAHOMA TOBACCO RESEARCH CENTER
STEPHENSON CANCER CENTER
ASSOCIATE PROFESSOR, DEPARTMENT OF FAMILY
AND PREVENTIVE MEDICINE
UNIVERSITY OF OKLAHOMA HEALTH SCIENCES CENTER
OKLAHOMA CITY, OK 73104

WHITT-GLOVER, MELICIA C, PHD
PRESIDENT AND CHIEF EXECUTIVE OFFICER
GRAMERCY RESEARCH GROUP, LLC
WINSTON-SALEM, NC 27106

WILLIAMS, KRISTINE N, BSN, PHD *
SALLY MATHIS HARTWIG PROFESSOR
GERONTOLOGICAL NURSING
COLLEGE OF NURSING
THE UNIVERSITY OF IOWA
IOWA CITY, IA 52242

WONG, FRANK Y, PHD
ASSOCIATE PROFESSOR
DEPARTMENT OF BEHAVIORAL SCIENCES
AND HEALTH EDUCATION
ROLLINS SCHOOL OF PUBLIC HEALTH
EMORY UNIVERSITY
ATLANTA, GA 30322

WU, BEI, PHD *
PAULINE GRATZ PROFESSOR OF NURSING AND
PROFESSOR OF GLOBAL HEALTH
DIRECTOR FOR INTERNATIONAL RESEARCH
SCHOOL OF NURSING
DUKE UNIVERSITY
DURHAM, NC 27710

MAIL REVIEWER(S)

CHRISTENSEN, KATHEE M, PHD
PROFESSOR
DEPARTMENT OF COMMUNICATIVE DISORDERS
SAN DIEGO UNIVERSITY
SAN DIEGO, CA 92182

KARNIK, NIRANJAN, MD, PHD
ASSOCIATE PROFESSOR
DEPARTMENT PSYCHIATRY
RUSH UNIVERSITY MEDICAL CENTER
CHICAGO, IL 60612

SMITH, TRISTRAM H, PHD
PROFESSOR
DEPARTMENT OF PEDIATRICS
SCHOOL OF MEDICINE AND DENTISTRY
UNIVERSITY OF ROCHESTER
ROCHESTER, NY 14618

SCIENTIFIC REVIEW OFFICER

BELLINGER, JESSICA, PHD
CENTER FOR SCIENTIFIC OF REVIEW
NATIONAL INSTITUTES OF HEALTH
BETHESDA, MD 20892

OLUFOKUNBI SAM, DELIA, PHD
SCIENTIFIC REVIEW OFFICER
CENTER FOR SCIENTIFIC REVIEW
NATIONAL INSTITUTES OF HEALTH
BETHESDA, MD 20892

EXTRAMURAL SUPPORT ASSISTANT

BARTLETT, VALERIE
EXTRAMURAL SUPPORT ASSISTANT
CENTER FOR SCIENTIFIC REVIEW
NATIONAL INSTITUTES OF HEALTH
BETHESDA, MD 20892

* Temporary Member. For grant applications, temporary members may participate in the entire meeting or may review only selected applications as needed.

Consultants are required to absent themselves from the room during the review of any application if their presence would constitute or appear to constitute a conflict of interest.
