# Supplementary material for: Understanding the Impact of Childhood Sexual Abuse on Men’s Risk Behavior: Protocol for a Mixed-Methods Study
Source: JMIR Res Protoc. 2018 Feb 26;7(2):e62. doi: 10.2196/resprot.9071 (PMC5847822; doi:10.2196/resprot.9071)
Supplement: Multimedia Appendix 2 [file resprot_v7i2e62_app2.pdf]

**Construct**	**Social identity and group membership**	**Assessment (M, IG)***
Gender	Gender identity and expression<br>Identification with cultural masculine norms	M1a - M1b<br>M13, IG7
Sexual orientation	Self-identification (How do you see yourself?)<br>Sexual attraction, behavior, and fantasies<br>Emotional, social, and lifestyle preferences	M2, IG4, IG8
Race and ethnicity	Racial / ethnic identity, cultural estrangement	M1d - M1e, M12, IG6
**Factors hypothesized to be influenced by the intersection of social identities:**		
• Appraisal and interpretation of childhood sexual experiences		M6a – M6d, IG1 – IG3, IG5
• Disclosure and concealment of same-sex behavior		M4, IG7
• Psychological distress and emotional functioning		M9 – M11, IG9
• Current and past substance use		M7 – M8, IG9, IG10
• Access to substance abuse treatment or other health services		IG11 – IG13

*Note: The figure indicates quantitative measures (M) and interview guide topics (IG) that will be used to examine each of the social identities and factors hypothesized to be influenced by the intersection of these identities. See Tables 2 and 3 for specific M and IG.
