# Supplementary material for: Understanding the Impact of Childhood Sexual Abuse on Men’s Risk Behavior: Protocol for a Mixed-Methods Study
Source: JMIR Res Protoc. 2018 Feb 26;7(2):e62. doi: 10.2196/resprot.9071 (PMC5847822; doi:10.2196/resprot.9071)
Supplement: Multimedia Appendix 3 [file resprot_v7i2e62_app3.pdf]

**Aim 1**	(1) childhood sexual experience (CSE) with a male (i.e., ages of participant and partner, relationship to partner, frequency, where and how the encounter(s) occurred, sexual behavior, degree of coercion); (2) CSE with a female; (3) appraisal of CSE with male and female partners (i.e., desired, unwanted, or considered abusive by the participant) *and why*; for participants who appraise their sexual experience(s) as unwanted, coercive or abusive, when they first recognized this.
**Aim 2**	(4) how men recall coming to terms with their sexual identity; (5) changes over time in perceptions of CSE; (6) how cultural and societal attitudes toward CSE influence appraisal and disclosure of abuse; (7) identification with masculinity norms (e.g., emotional control, homophobia, shame), and how these norms influence concealment and disclosure of CSE and same-sex behavior; (8) current sexual relationships and behaviors with men and women, and perceptions of whether CSE (and appraisals) have influenced their adult relationships (e.g., problems with intimacy, revictimization); (9) how men have coped with CSE, particularly unwanted or abusive experiences; (10) when and how men initiated drug and alcohol use.
**Aim 3**	(11) prior experiences with alcohol and drug treatment, risk reduction interventions, mental health services, and other health care settings; (12) whether participants perceive a need for the inclusion of CSE histories in such services and settings; (13) what specific types of services and interventions they perceive as needed to address abusive CSEs of Black and Latino MSM and MSMW; (14) what intervention format they would find most appealing or acceptable; (15) specific times, days, or frequencies in which the intervention would be welcome or unsuitable.
